# Outbreak of *Trichinella* T9 Infections Associated with Consumption of Bear Meat, Japan

**DOI:** 10.3201/eid2408.172117

**Published:** 2018-08

**Authors:** Katsushige Tada, Hiromichi Suzuki, Yosuke Sato, Yasuyuki Morishima, Isao Nagano, Haruhiko Ishioka, Harumi Gomi

**Affiliations:** Mito Kyodo General Hospital, University of Tsukuba, Mito, Japan (K. Tada, H. Ishioka, H. Gomi);; Tsukuba Medical Center Hospital, Tsukuba, Japan (H. Suzuki);; Ibaraki Prefecture Mito Health Center, Mito (Y. Sato);; National Institute of Infectious Diseases, Tokyo, Japan (Y. Morishima);; Gifu University, Gifu, Japan (I. Nagano)

**Keywords:** Trichinella T9, bear meat, Japan, trichinellosis, outbreak, foodborne disease, parasites

## Abstract

An outbreak of trichinellosis occurred in Japan in December 2016. All case-patients had eaten undercooked bear meat, from which *Trichinella* larvae were subsequently isolated. DNA sequencing analysis of the mitochondrial genes cytochrome *c*-oxidase subunit 1 and internal transcribed spacer 2 confirmed that *Trichinella* T9 had caused the outbreak.

Trichinellosis is a parasitic disease caused by the *Trichinella* spp. nematode that is contracted by eating raw or undercooked meat from infected animals. Approximately 100 species of animals, including humans, can be infected (*1*). The most common source of human trichinellosis is meat from pigs or wild boar. A total of 65,818 human cases were reported from 41 countries during 1986–2009 (*2*).

In Japan, trichinellosis is rarely encountered in the clinical setting, and only 5 imported cases (1 in 1998 [*3*], 1 in 1999 [*4*], 1 in 2003 [*5*], 2 in 2009 [*6*]) have been reported during the past few decades. Three outbreaks of domestically acquired trichinellosis have been reported since 1975 (*7*), the last reported outbreak occurring in 1981; all were associated with bear meat consumption, but the etiologic agents were not identified at the species level. Since then, no outbreaks were reported until late 2016.

## The Study

**Figure 1:**
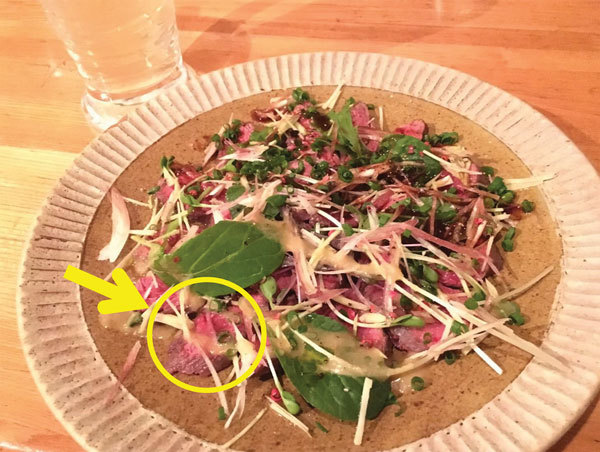
Bear meat dish implicated in an outbreak of *Trichinella* T9 infection, Japan, December 2016. Bear meat slices are marked with a circle and an arrow.

In December 2016, a previously healthy young man was referred to Tsukuba Medical Center Hospital (Tsukuba, Ibaraki Prefecture, Japan) for a fever, rash, malaise, and eosinophilia. He claimed that he had eaten a bear meat dish (Figure 1) at a restaurant in Mito, Ibaraki Prefecture, Japan, with his 4 friends, who all had similar signs and symptoms. Subsequently, a total of 32 patients who had consumed the bear meat were reported to the Ibaraki Prefecture Mito Health Center; 28 patients had been evaluated at hospitals. Ethics approval for this research was obtained from the Institutional Review Board of Mito Kyodo General Hospital, University of Tsukuba, Mito, Japan (No. 16-69). All patients provided informed consent for their data to be included in this study.

Among the 28 patients who underwent evaluation, 21 had signs and symptoms that were compatible with trichinellosis. Each serum sample obtained from the 28 patients was tested 3 times for antibodies to *Trichinella* spp., as previously described (*6*). We performed antibody titer testing with ELISA using excretory–secretory (ES) antigens from *Trichinella spiralis* at the patient’s initial presentation and >2 weeks after the first serum samples were obtained (Technical Appendix). We defined a confirmed case as illness in a patient with a history of consuming raw bear meat, clinical symptoms compatible with trichinellosis, and serologic evidence of trichinellosis. A probable case was defined as illness in a patient with a history of consuming raw bear meat, clinical symptoms compatible with trichinellosis, and a negative serologic test result (*8*).

In total, 19 (90.4%) patients, all symptomatic, had an antibody titer higher than the cutoff (Table 1); 2 symptomatic patients had an antibody titer lower than the cutoff (titer <200 on convalescent serologic evaluations). All 7 asymptomatic patients had negative serologic test results. Consequently, we identified 21 trichinellosis patients in our study, representing 19 confirmed and 2 probable cases.

**Table 1:** Serologic test results for 28 patients who consumed bear meat associated with *Trichinella* T9 infection, Japan, December 2016*

Patient no.	Signs and symptoms	Highest blood eosinophil count, cells/L	Initial serologic test titer	No. days postinfection	Convalescent-phase serologic test titer	No. days after first blood collection
1	Yes	7.1 × 10^9^	<200	23	6,400	24
2	Yes	4.3 × 10^9^	<200	23	12,800	19
3	Yes	2.7 × 10^9^	<200	27	1,600	20
4	Yes	10.1 × 10^9^	<200	23	800	19
5	Yes	7.8 × 10^9^	800	24	3,200	17
6	Yes	8.8 × 10^9^	<200	25	1,600	17
7	Yes	3.1 × 10^9^	<200	25	1,600	17
8	Yes	1.9 × 10^9^	800	25	6,400	18
9	Yes	11.1 × 10^9^	<200	25	3,200	18
10	Yes	2.4 × 10^9^	<200	24	3,200	15
11	Yes	5.0 × 10^9^	400	12	3,200	16
12	Yes	4.2 × 10^9^	<200	13	800	16
13	Yes	8.5 × 10^9^	200	22	6,400	14
14	Yes	5.3 × 10^9^	<200	20	800	15
15	Yes	10.8 × 10^9^	200	20	3,200	14
16	Yes	2.9 × 10^9^	<200	23	<200	24
17	Yes	4.3 × 10^9^	<200	21	<200	16
18	Yes	1.9 × 10^9^	<200	20	400	13
19	No	0.1 × 10^9^	<200	22	<200	29
20	No	0.2 × 10^9^	<200	18	<200	15
21	No	0.1 × 10^9^	<200	19	<200	28
22	No	0.1 × 10^9^	<200	27	<200	24
23	Yes	2.3 × 10^9^	<200	23	6,400	15
24	Yes	2.9 × 10^9^	<200	23	400	16
25	No	0.4 × 10^9^	<200	25	<200	31
26	No	0.2 × 10^9^	<200	21	<200	14
27	No	0.1 × 10^9^	<200	11	<200	29
28	Yes	2.0 × 10^9^	<200	27	400	13

*ELISA was performed to detect *Trichinella* antigens. The cutoff point (0.148) was 3 times the mean value of A_414_ from the negative serum sample of 100 healthy persons. Of the 28 patients evaluated, 21 had signs and symptoms compatible with trichinellosis. Patients 1–15 also had elevated antibody titers; however, the antibody titers of patients 16 and 17 were not elevated. These cases were defined as probable trichinellosis, as previously described (*8*).

**Figure 2:**
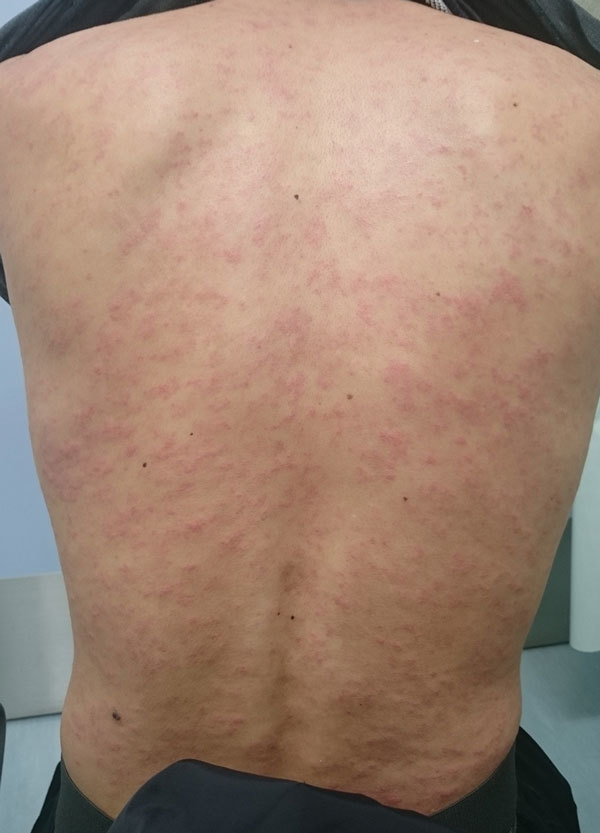
Rash on the back of a patient (patient 10 in Table 1) with confirmed *Trichinella* T9 infection associated with consumption of bear meat, Japan, December 2016. Patient had onset of macular and papular, confluent, and pruritic rash with diffuse blanching on the scalp, face, chest, abdomen, back, and upper and lower extremities. Photo taken 24 days after the patient had consumed the implicated bear meat.

We compiled and assessed demographic and clinical data on the 21 patients with confirmed and probable trichinellosis (Table 2). Median age was 35 years (range 23–58 years); 10 (48%) patients were female and 11 (52%) male. Thirteen patients (62%) had consumed >3 slices of infected bear meat (≈10 g per slice). The median incubation period was 19 days (range 6–34 days). All patients had a rash (Figure 2), 20 (95%) had a fever, 17 (81%) had myalgia, 10 (48%) had facial edema, and 9 (43%) had peripheral edema. Only 5 (24%) patients had diarrhea (range of onset day 1–16 of illness) during the outbreak. Nine (43%) patients had conjunctivitis, and 2 (10%) had uveitis. 

**Table 2:** Epidemiologic, clinical, and laboratory data for 21 symptomatic patients with probable or confirmed *Trichinella* T9 infection, Japan, December 2016*

Characteristic	Value
Median age, y (range)	35 (23–58)
Sex	
F	10 (48)
M	11 (52)
Consumed >3 slices of infected bear meat	13 (62)
Median incubation period, d (range)	19 (6–34)
Signs and symptoms	
Fever	20 (95)
Rash	21 (100)
Myalgia	17 (81)
Fatigue	9 (43)
Facial edema	10 (48)
Peripheral edema	9 (43)
Diarrhea	5 (24)
Conjunctivitis	9 (43)
Uveitis	2 (10)
Median duration from date of eating bear meat to date of blood sampling, d (range)	23 (12–27)
Median leukocyte count, cells/L (range)	7.2 × 10^9^ (3.9 × 10^9^ to 16.9 × 10^9^)
Median eosinophil count, cells/L (range)	1.0 × 10^9^ (0.1 × 10^9^ to 4.3 × 10^9^)
Median aspartate aminotransferase level, IU/L (range)	24 (12–41)
Median alanine aminotransferase level, IU/L (range)	22 (9–73)
Median creatine kinase level, IU/L (range)	147 (57–786)
Median C-reactive protein level, mg/L (range)	9.4 (0.4–67.5)

*Laboratory data were obtained at initial presentation. Values are no. (%) patients except as indicated.

At the time of initial evaluation, the median eosinophil count was 1.0 × 10^9^/L (range 0.1 × 10^9^/L to 4.3 × 10^9^/L), and the median creatine kinase level was 147 IU/L (range 57–786 IU/L). All patients were treated with albendazole (200 mg or 400 mg, 2×/d for 10–14 days), with or without prednisolone. In 1 case, albendazole was changed to mebendazole because of a mild increase in the patient’s aspartate aminotransferase and alanine aminotransferase levels, which was later considered to have occurred because of trichinellosis itself. None of the patients had serious complications of trichinellosis or major adverse events during treatment.

The bear meat came from a brown bear (*Ursus arctos*) that had been hunted in Hokkaido Prefecture in November 2016. The meat had been divided into 3 blocks that were preserved in cold storage. Two of these blocks were eaten during this outbreak. The first bear meat block was brought into a restaurant in Mito. In late November, it was seared and served in thin slices with herbs (Figure 1; Technical Appendix Figure). This bear meat was kept in cold storage and served for 2 days, after which it was preserved in a freezer. The temperature of the cold storage and the freezer were not recorded. Japan Industrial Standard (JIS B 8630) defines the temperature of refrigerated storage as not below 0°C and freezing as <–20°C. The bear meat was served after being reheated for a few minutes. The second bear meat block was cooked steak-style to a medium-rare condition; 1 of the patients had eaten meat from this block (online Technical Appendix Figure). The third bear meat block was stored in a freezer without being consumed. We used this meat for the analysis of *Trichinella* spp., which was performed at the National Institute of Infectious Diseases (Tokyo, Japan).

We artificially digested the bear meat with 0.5% pepsin-0.8% HCl solution and then performed a microscopic examination on the sediment. We detected encapsulated larvae with a distinctive esophageal structure (stichosome). The density of the larvae was 84 larvae/g. For the molecular identification of the larvae, we amplified cytochrome *c*-oxidase subunit 1 (*cox1*) and internal transcribed spacer 2 (ITS2) by using PCR with primer pairs described by Kanai et al. (*9*). A subsequent sequence analysis showed that both sequences (GenBank accession nos. LS361217 for *cox1* and LS361216 for ITS2) were identical to the corresponding sequences of *Trichinella* T9 (GenBank accession nos. KM357420 for *cox1* and AB255886 for ITS2).

Nine species (*T. spiralis, T. britovi, T. nativa, T. nelsoni, T. murrelli, T. zimbabwensis, T. papuae, T. pseudospiralis, T. patagoniensis*) and 3 unclassified genotypes (T6, T8, and T9) are currently recognized in the genus *Trichinella* (*10*). Among them, *T. spiralis* is the most common species in the world (*11*). The taxonomic status of *Trichinella* species in Japan has not yet been fully elucidated. A recent molecular study revealed that the *Trichinella* isolates obtained from animal specimens in Japan included *Trichinella* T9 (*12*) and *T. nativa* (*13*), but *T. spiralis* has not yet been found in Japan (*9*). *Trichinella* T9 has only been reported in Japan. Therefore, it is considered to be native to Japan. *Trichinella* T9 has been detected and confirmed in a brown bear (*13*), raccoons (*14*), raccoon dogs (*13*,*14*), and red foxes (*13*), but no cases of human infection have been reported.

In this outbreak, 2 symptomatic patients and 7 asymptomatic patients had negative serologic test results. A second blood specimen was collected from these patients 33–56 days after the consumption of the bear meat. According to the pertinent literature data (*15*), serum conversion has been observed up to 65 days postinfection. Thus, we need to consider the possibility of a delay in serum conversion for these 9 patients.

## Conclusions

We describe an outbreak of trichinellosis that occurred because of the consumption of bear meat infected with *Trichinella* T9. Public awareness should be raised and education should be promoted to prevent further outbreaks of trichinellosis in Japan.

Technical Appendix Method for performing ELISA using excretory–secretory antigens from *Trichinella spiralis*.
