# Supplementary material for: Outbreak of Trichinella T9 Infections Associated with Consumption of Bear Meat, Japan
Source: Emerg Infect Dis. 2018 Aug;24(8):1532–5. doi: 10.3201/eid2408.172117 (PMC6056131; doi:10.3201/eid2408.172117)
Supplement: Technical Appendix — Method for performing ELISA using excretory–secretory antigens from Trichinella spiralis. [file 17-2117-Techapp-s1.pdf]

Article DOI: https://doi.org/10.3201/eid2408.172117

Outbreak of *Trichinella* T9 Infections Associated with Consumption of Bear Meat, Japan

Technical Appendix

Method for Performing ELISA Using Excretory–Secretory Antigens from *Trichinella spiralis*

ES products using as antigen for an ELISA were prepared from *T. spiralis* (ISS413) muscle-stage larvae according to the method of Gómez-Morales et al. (*1*). In brief, muscle-stage larvae were isolated by pepsin-HCl digestion from mice at 30 days post-infection. The larvae were washed 3 times in phosphate-buffered saline (PBS) and then incubated in Dulbecco's modified Eagle's medium at 37°C for 18 h. The medium was filtered through a 0.2-µm YM-5 filter and concentrated 100 times in an Amicon pressure concentrating chamber (Amicon, Inc., Billerica, MA, USA).

The ELISA was performed as reported in our previous study (*2*,*3*) with slight modifications. In brief, 96-well microtiter plates (MaxiSorp, Nalge Nunc International, Tokyo, Japan) were sensitized with ES antigen at 5 µg/ml in 0.05 M bicarbonate buffer, pH 9.6 (100 µl/well) for 3 h at 37°C and overnight at 4°C. After the microplates were washed 3 times with PBS, they were blocked with 150 µl of PBS containing 2% bovine serum albumin (BSA) for 2 h at 37°C. After washing with PBS-0.05% Tween 20, the microplates were probed with a diluted human serum sample (1:200–1:25,600, 100 µl/well) in PBS containing 1% BSA for 1 h at 37°C. After washing, 100 µl of 1:10,000-diluted goat anti-mouse IgG (Fab-specific) peroxidase-conjugate (Sigma Chemical Co., St. Louis, MO, USA) was incubated for 1 h at 37°C. For color development, 2–2'-azono-bis (3-ethylbenzthiazoline-6-sulfonic acid) (Sigma Chemical Co.) was added to each well as a substrate (0.3 mg/ml, 100 µl/well), and the reaction was terminated after 60 min by adding 50 µl of 1.25% sodium fluoride per well. The absorbance at 414 nm was monitored with a Multiskan JX plate reader (Labsystems, Helsinki, Finland). Since "3 × the A_{414} means of 1:200-diluted negative sera from 100 healthy persons" was larger than "the means plus 3 standard deviations," "3 × the A_{414} mean of the negative sera" was determined as the cutoff

value in further experiments, resulting in a value of 0.148 for our ELISA test. Each plate contained four positive and four negative reference serum samples. Three-well repeats were measured for each diluted serum sample. The ELISA titers were shown as the highest serum dilution that yielded an A_{414} greater than the cutoff point.

References

1. Gómez-Morales MA, Ludovisi A, Amati M, Cherchi S, Pezzotti P, Pozio E. Validation of an ELISA for the diagnosis of human trichinellosis. Clin Vaccine Immunol. 2008;15:1723–9. PubMed http://dx.doi.org/10.1128/CVI.00257-08

2. Nagano I, Pei F, Wu Z, Wu J, Cui H, Boonmars T, et al. Molecular expression of a cysteine proteinase of *Clonorchis sinensis* and its application to an enzyme-linked immunosorbent assay for immunodiagnosis of clonorchiasis. Clin Diagn Lab Immunol. 2004;11:411–6. PubMed

3. Lo YC, Hung CC, Lai CS, Wu Z, Nagano I, Maeda T, et al. Human trichinosis after consumption of soft-shelled turtles, Taiwan. Emerg Infect Dis. 2009;15:2056–8. PubMed http://dx.doi.org/10.3201/eid1512.090619

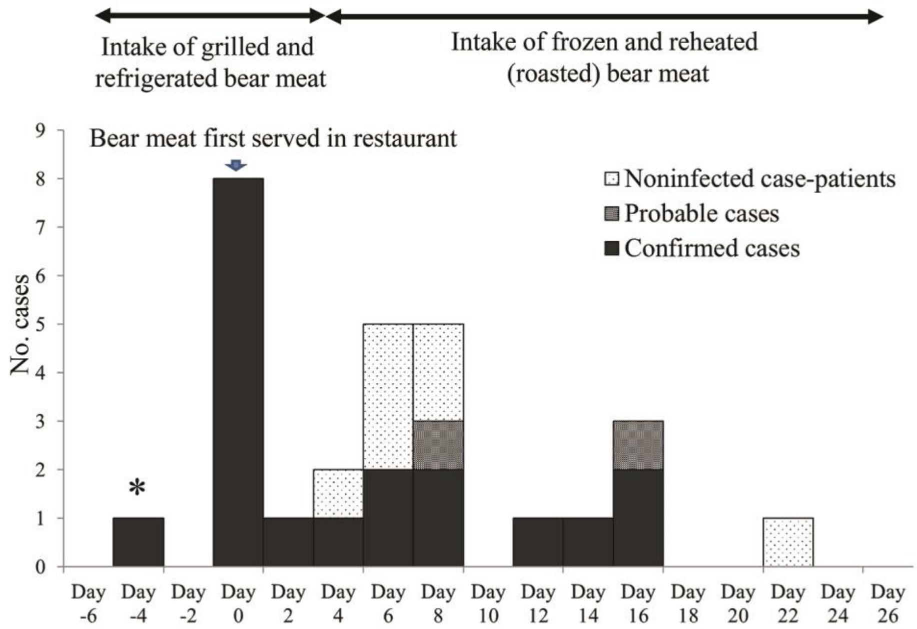

Technical Appendix Figure. The relationship between the date on which the infected bear meat was consumed and the occurrence of trichinellosis. A confirmed case was defined as patients with 1) a history of consuming raw bear meat; 2) clinical symptoms compatible with trichinellosis; and 3) serologic evidence of trichinellosis. A probable case was defined as patients with 1) a history of consuming raw bear meat; 2) clinical symptoms compatible with trichinellosis; and 3) negative serologic results. Asterisk indicates that the patient consumed bear meat from a different block of meat from the other case-patients.
